# The Axe-Txe Complex of *Enterococcus faecium* Presents a Multilayered Mode of Toxin-Antitoxin Gene Expression Regulation

**DOI:** 10.1371/journal.pone.0073569

**Published:** 2013-09-03

**Authors:** Lidia Boss, Łukasz Labudda, Grzegorz Węgrzyn, Finbarr Hayes, Barbara Kędzierska

**Affiliations:** 1 Department of Molecular Biology, University of Gdańsk, Gdańsk, Poland; 2 Faculty of Life Sciences and Manchester Institute of Biotechnology, the University of Manchester, Manchester, United Kingdom; University of Illinois at Chicago College of Medicine, United States of America

## Abstract

Multidrug-resistant variants of human pathogens from the genus *Enterococcus* represent a significant health threat as leading agents of nosocomial infections. The easy acquisition of plasmid-borne genes is intimately involved in the spread of antibiotic resistance in enterococci. Toxin-antitoxin (TA) systems play a major role in both maintenance of mobile genetic elements that specify antibiotic resistance, and in bacterial persistence and virulence. Expression of toxin and antitoxin genes must be in balance as inappropriate levels of toxin can be dangerous to the host. The controlled production of toxin and antitoxin is usually achieved by transcriptional autoregulation of TA operons. One of the most prevalent TA modules in enterococcal species is *axe-txe* which is detected in a majority of clinical isolates. Here, we demonstrate that the *axe-txe* cassette presents a complex pattern of gene expression regulation. Axe-Txe cooperatively autorepress expression from a major promoter upstream of the cassette. However, an internal promoter that drives the production of a newly discovered transcript from within *axe* gene combined with a possible modulation in mRNA stability play important roles in the modulation of Axe:Txe ratio to ensure controlled release of the toxin.

## Introduction

Recent analyses of the dynamics of invasive infections causing bacteraemia in European countries showed the fastest increase in the number of infections caused by *Enterococcus* sp. relative to other tested pathogens [1]. The treatment of infections caused by these bacteria is particularly difficult because of their intrinsic resistance to certain groups of antibiotics including penicillins, cephalosporins, and aminoglycosides. Moreover, the tendency of enterococci to acquire and exchange a wide variety of resistance determinants through horizontal transfer of mobile genetic elements such as plasmids and transposons further reduces the antibiotics available to treat certain enterococcal infections [2,3].

Molecular mechanisms responsible for the spread and stable maintenance of antibiotic resistance genes located on plasmids are well documented for model bacteria such as *Escherichia coli*. One of the stabilisation mechanisms that assures effective propagation of low copy number bacterial plasmids is their active segregation to daughter cells during cell division. Additionally, plasmids encode toxin-antitoxin (TA) systems that act in postsegregational killing of cells that have failed to acquire a plasmid at division [4]. In these daughter cells devoid of a plasmid, the degradation of antitoxin and the lack of its *de novo* synthesis leads to the release of the toxin which interacts with its intracellular target, leading to cell death or inhibition of metabolic processes. Thus, as progeny die if the plasmid is lost, bacteria become “addicted” to TA modules located on plasmids. TA complexes are also widely encoded by chromosomes of prokaryotes. Here, the toxin is activated in response to diverse stress and nutritional stimuli that result in downregulation of metabolism and/or programmed cell death. Chromosomal TAs are also implicated in antibiotic persistence, biofilm formation, and bacteriophage resistance [5].

To date, five different TA types based on the nature and mode of action of the antitoxin have been proposed [6]. Our current study focuses on type II TA systems, in which both the toxin and the antitoxin are proteins. In this group, TA modules generally have similar organizations and modes of expression regulation [5,7–9]. The cassettes usually consist of a pair of genes forming an operon. The first gene encodes a more labile antitoxin which is a target for Clp or Lon proteases, whereas the second gene specifies a stable toxin. Strong and specific interactions between toxin and antitoxin proteins, as well as precise transcriptional regulation of their expression, are characteristic feature of TA complexes. Expression of the two genes must be in balance as inappropriate levels of toxin can be dangerous to the host. The controlled production of toxin and antitoxin is achieved by transcriptional regulation of TA operons. Usually, type II TA operons are negatively autoregulated at the transcriptional level, but the detailed molecular mechanisms that underpin this process are still poorly understood for most TA modules. Nevertheless, a common pattern involves binding of the antitoxin to palindromic sequences in the promoter region by its N-terminal domain, making the antitoxin the principal factor for transcriptional repression. The C-terminal domain of the antitoxin generally binds to the toxin which acts as a co-repressor by increasing the affinity and stability of the regulatory complex. This canonical pattern of transcriptional autoregulation characterizes the best described type II TA cassettes, including YefM-YoeB, RelBE, MazEF, CcdAB and Kis-Kid [10–14]. Additionally, cooperative binding of certain TA complexes to operator DNA occurs only when toxins and antitoxins are in proper stoichiometric relationships. Excess toxin stimulates operon transcription by releasing the TA complex from the operator site which prevents uncontrolled toxin activation [15,16].

Nevertheless, some exceptions to this general pattern of type II TA regulation are known. Binding of the antitoxin alone is sufficient for full repression of the *parDE* TA operon on low copy number plasmid RK2 [17]. Additional genes are involved in repression of the *paaR-paaA-parE* and ε*-ζ-ω* TA systems. In the case of the PaaA antitoxin-ParE toxin complex in *E. coli* O157:H7, it autorepresses the main promoter only partially, but the PaaR protein is needed for full down-regulation of transcription [18]. On the other hand, in the case of the ε-ζ-ω system of plasmid pSM19035, the ζ toxin and ε antitoxin have no roles in transcriptional control. Instead, transcription of the operon is efficiently repressed solely by the ω protein [19]. Unlike its *E. coli* homologues, the chromosomal type II *mazEF* operon of *Staphylococcus aureus* is not autoregulated. Instead, the global transcriptional regulator SarA activates the cassette, whereas the alternative sigma factor σ^B^ represses its transcription, probably indirectly [20].

As TAs are key for both maintenance of mobile genetic elements that specify antibiotic resistance and in bacterial persistence and virulence, dissection of these systems in pathogenic bacteria, including enterococci, is crucial [21]. Par and Axe-Txe encoded by plasmids of *Enterococcus faecalis* and *E. faecium*, respectively, were among the first TA systems identified in enterococci [22–24]. The *par* locus specifies two small RNA molecules, RNA I and RNA II. The former is translated into a 33 amino acid toxic peptide whose expression is regulated posttranscriptionally by RNA II [25]. Differential decay patterns of RNA I and RNA II elicit translation of the former in plasmid-free cells. The toxin disrupts cell membrane function by an as yet unknown mechanism [26].

The type II *axe-txe* module was first identified on the multidrug resistant pRUM plasmid from a clinical isolate of *E. faecium*. Axe-Txe is a plasmid maintenance complex not only in enterococci, but also in evolutionary diverged species, including *Bacillus* sp. and *E. coli*. Axe-Txe is homologous to the YefM-YoeB complex of *E. coli* [24]. Txe (85 amino acids) is a positively charged toxin that is neutralized by Axe (89 amino acids), a negatively charged antidote. When liberated from the complex, Txe acts as an endoribonuclease that cleaves cellular mRNA downstream of AUG start codons [27]. Txe thereby inhibits bacterial growth and cell division [24]. Axe-Txe and certain other TA modules are found widely in antibiotic resistant enterococci, including vancomycin resistant isolates [28–30].

In this study, we investigated mechanisms underpinning regulation and expression control of the *axe-txe* module. Our studies show that the expression of *axe*-*txe* genes is different than in other described TA systems. Notably, an internal promoter that drives the production of a novel transcript was detected within the *axe* gene. This message, together with mRNA stability control, may be a part of a complex regulatory circuit that tunes the ratio of Axe antitoxin to Txe toxin.

## Materials and Methods

### Strains

*E. coli* DH5α was used for plasmid construction and Rosetta(DE3) for crude extract preparation with Axe and Axe-Txe overproduction from pET22axe and pET22at_axe-txe, respectively. Strain SC301467 [31] was used for DNA and RNA isolation and for luminescence assays, and C600*polA1* was used in plasmid stability assays. Bacteria were grown in Luria-Bertani (LB) medium at 37°C. Ampicillin and chloramphenicol were added to final concentrations of 100 and 34 or 10 µg/ml, respectively, when required.

### Plasmids and oligonucleotides

Oligonucleotides and plasmids used in this study are listed in Tables 1 and 2, respectively.

**Table 1:** Oligonucleotides used in this study.

**Oligonucleotide**	**Sequence (5’–3’)**
1	GACG A A T T CTACAATTTCAGGTGGCAC
2	GGTG A A T T CGTAAACTTGGTCTGACAG
3	CCGATTAC A T A T GGAAGCAGTAGCTTATTC
4	GAC T C G A GATCATCAGATTCAACCTCG
5	TTCAG G A T C CAGGATTATGTGTATTGCG
6	CCGCA A G C T TTTAAGTTTCTGACCCTTTCC
7	GAGTA C T A G TGAAAAAGCAGGATTTGAGG
8	CCAAG G A T C CGAATAAGCTACTGCTTCC
9	CGGTCG G A T C CAATAAAGATAATCATC
10	ATTCG G A T C CTTAATAGTGATCTTTTGCAG
11	CGGGA C T A G TTAGAAATAAATAAGGGGT
12	CAAAAAGAGATTACGACTCTATGCAAGAAACG
13	CGTTTCTTGCATAGAGTCGTAATCTTTTTTG
14	CGCGGG A A T T CTAGAAATAAATAAGGGGT
15	GCACTAAATCATCACTTTCGGGAAAG
16	GAGTG A A T T CGAAAAAGCAGGATTTGAGG
17	ATCG G A T C CGTAATACGCGTAAC
18	CCGCA A G C T TGCTCATGCCAATAAAGATAATC
19	[BTN]AGCAACTAAAGCAGAAGTACGGC
20	TCATATAACTACGTAAATTTTGGCGG
21	[BTN]TTCCGCCAAAATTTACGTAGTTA
22	TTGCATAGAATCATAATCTCTTTTTGA

Restriction sites or introduced mutations are underlined.

**Table 2:** Plasmids used in this study.

**Name**	**Description**	**Reference**
pBBRlux	Vector for generating transcriptional fusion to *lux*, Cm^r^	[32]
pBBRlux-amp	Vector for generating transcriptional fusion to *lux, bla* gene was amplified with primers 1/2 and cloned into EcoRI site within *cat* gene	This study
pET22b(+)	IPTG-inducible expression vector allowing fusion of C-terminal His_6_ tag to the target protein, Amp^R^	Novagen
pET22axe	*axe* gene amplified with primers 3/4, digested with NdeI-XhoI and cloned between equivalent sites in pET22(+)	This study
pET22at_axe-txe	*at_axe-txe* fragment amplified with primers 5/6, digested with BamHI-HindIII and cloned between equivalent sites in pET22(+)	This study
pluxat	*p*_*at*_ promoter-operator region amplified with primers 7/8 (209 bp), digested with SpeI-BamHI and cloned between equivalent sites in pBBRlux-amp	This study
pluxat_axe	fragment containing *p*_*at*_ promoter-operator region and *axe* gene amplified with primers 7/9 (497 bp), digested with SpeI-BamHI and cloned between equivalent sites in pBBRlux-amp	This study
pluxat_axe-txe	fragment containing *p*_*at*_ promoter-operator region and *axe-txe* genes amplified with primers 7/10 (708 bp), digested with SpeI-BamHI and cloned between equivalent sites in pBBRlux-amp	This study
pluxaxe	*p*_*axe*_ promoter-operator region amplified with primers 9/11 (353 bp), digested with SpeI-BamHI and cloned between equivalent sites in pBBRlux-amp	This study
pluxaxemut	*p*_*axe*_ promoter-operator region with mutated -10 box (site-directed mutagenesis with primers 12/13) amplified with primers 9/11 (353 bp), digested with SpeI-BamHI and cloned between equivalent sites in pBBRlux-amp	This study
pluxaxe-txeW5C	*axe-txe* genes with amino acid change in Txe protein (W5C) amplified with primers 10/11 (564 bp), digested with SpeI-BamHI and cloned between equivalent sites in pBBRlux-amp	This study
pREG531	pFH450 derivative plasmid containing *axe-txe* cassette, used for amplifications of this module and plasmid stability tests, Cm^r^	[24]
pREGpaxemut	pREG531 derivative with *p*_*axe*_ promoter-operator region mutated in -10 box (site-directed mutagenesis with primers 12/13)	This study
pREGΔaxetxe	pREG531 derivative, where axe-txe cassette was cut out with enzymes KpnI and SpeI and vector was religated	This study
pTE103	Vector for generating transcription templates, contains the multicloning site from pUC8 placed upstream from a bacteriophage T7 transcriptional terminator, Amp^R^	[33]
pTEat_axetxe	fragment containing *p*_*at*_ promoter-operator region and *axe-txe* genes amplified with primers 6/16, digested with EcoRI-HindIII and cloned between equivalent sites in pTE103	This study
pTEat_axetxemut	fragment containing *p*_*at*_ promoter-operator region and *axe-txe* genes with mutated -10 box in *p*_*axe*_ promoter amplified with primers 6/16, digested with EcoRI-HindIII and cloned between equivalent sites in pTE103	This study
pTEaxetxeW5C	*axe-txe* genes with amino acid change in Txe protein (W5C) amplified with primers 6/14, digested with EcoRI-HindIII and cloned between equivalent sites in pTE103	This study
pTEaxe	*axe* and first 60 bp of *txe* genes amplified with primers 14/18, digested with EcoRI-HindIII and cloned between equivalent sites in pTE103	This study
pTEat_axe-txe_ter	fragment containing *p*_*at*_ promoter-operator region and *axe-txe* genes along with the terminator region downstream of *txe*, amplified with primers 16/17, digested with EcoRI-BamHI and cloned between equivalent sites in pTE103	This study

### Crude extract preparation

Bacteria were grown at 37°C in 10 ml of LB medium with appropriate antibiotic until OD_600_ ~0.5. Expression of *axe* (pET22axe) or *axe-txe* (pET22at_axe-txe) was induced with 1 mM IPTG and incubation continued for 3 hours. Cells were harvested at 1600 *g* for 10 min. The pellet was resuspended in 1 ml of buffer comprising 20 mM Tris–HCl pH 7.5 and 50 mM NaCl. The cells were sonicated and then centrifuged for 30 min at 15500 *g* at 4^o^C. Supernatant was dialysed against the same buffer containing 10% glycerol. The samples were aliquoted and stored at -20^o^C.

### Promoter fusion studies and bioluminescence assays

Strain SC301467 harbouring derivatives of pBBR*lux*-amp with the *lux* operon under transcriptional control of fragments containing different elements of *axe-txe* operon were used. PCR fragments were cloned into pBBR*lux*-amp between SpeI-BamHI restriction sites upstream of the promoterless *luxCDABE* to yield the transcriptional fusions *p*_*at*_::*lux* (primers 7/8)*, p*_*at*_ *axe::lux* (primers 5/7)*, p*_*at*_ *axe-txe::lux* (primers 7/10)*, p*_*axe*_ *::lux* (primers 9/11) and *p*_*axemut*_ *::lux* (primers 9/11). Overnight cultures carrying recombinant plasmids were diluted (1:100) into fresh LB medium and grown until OD_600_ ~0.4. Then luminescence of 200 µl of cells was measured in a luminometer (Berthold Technologies, Junior). Results in relative light units (RLU) were divided by the optical density (OD_600_) of the cultures.

### Plasmid stability assays

The bacteria containing different constructs were grown under selective conditions overnight. 10 µl of the resulting culture were used to inoculate 10 ml of fresh medium again with antibiotic pressure and left to grow with shaking for 12 hours. Next, 1/10000 dilutions were made every 12±3 hours in fresh medium without selective pressure. Successive subcultures were repeated 5 times in total. Samples from each subculture were plated on LB agar without antibiotic to obtain single colonies. For determination of plasmid stability one hundred colonies of each strain were streaked on LB agar plates supplemented with chloramphenicol and, as a control, to LB agar plates containing no antibiotic. The retention of chloramphenicol-resistance phenotype was shown as a percentage.

### Primer extension analysis

The promoters in the *axe-txe* cassette region were mapped with a ^32^P-labeled primer (primer 15) that anneals to the *lux* gene downstream from the region of interest. Total cellular RNA from strain SC301467 harbouring pBBRlux–based plasmids possessing transcriptional fusions of *p*_*at*_ or *p*_*axe*_ promoter-operator regions to the *lux* operon (pluxat or pluxaxe) were combined with the labeled primer. Primer extension reactions were done in total volumes of 10 µl containing 10 µg RNA, 0.6 pmol of labeled primer, RevertAid *H* *Minus* Reverse Transcriptase buffer (50 mM Tris-HCl pH 8.3, 50 mM KCl, 4 mM MgCl_2_, 10 mM DTT), 1 mM of each dNTPs, 10 U RiboLock RNase Inhibitor. Samples were denatured at 99^0^C for 2 min, and then incubated at 50^0^C for 1 hour. Next, 0.5 µl of 200 U/µl RevertAid *H* *Minus* Reverse Transcriptase (Fermentas) were added and samples were incubated at 42^0^C for 30 min. 5 µl of loading dye (95% formamide, 0.05% bromophenol blue, 0.05% xylene cyanol) were added and samples were denatured for 10 min at 99^0^C prior loading on a 6% sequencing gel along with sequencing reactions performed with the same labeled primer and appropriate plasmid DNA (SequiTherm EXCEL™ II DNA Sequencing Kit, Epicenter) according to the protocol.

### Electrophoretic mobility shift assays (EMSA)

5’-biotinylated, double-stranded PCR fragments that included the *p*_*at*_ (primers 19/20) and *p*_*axe*_ (primers 21/22) regulatory regions were used in EMSA. Reactions containing 0.1 nM of biotin–labeled DNA and bacterial crude extract at concentrations of 0, 1.25, 2.5, 5, 10, 12.5 and 25 µg/ml total protein were assembled in binding buffer (10 mM Tris-HCl pH 7.5, 50 mM NaCl, 1 mM DTT, 5 mM MgCl_2_, 1 µg of poly(dIdC), 2.5% glycerol) in final volumes of 20 µl and incubated for 20 min at 22°C. Then samples were electrophoresed on 6% native polyacrylamide gels in 0.5x TBE buffer for 120 min at 100V at 4°C. DNA was transferred by electroblotting to positively–charged nylon membrane (Millipore), and the transferred DNA fragments were immobilized onto the membrane by ultraviolet cross-linking. Detection of the biotin–labeled DNA was performed using the LightShift^TM^ chemiluminescent EMSA kit (Pierce).

### In vitro transcription analysis

Transcription activity within the *axe-txe* operon was analysed in multiround *in vitro* transcription assays performed on circular plasmid DNAs (derivatives of pTE103 vector) as indicated on figures. Reactions were done at 37^0^C in total volumes of 17 µl containing 40 mM Tris-HCl pH 8.0, 150 mM KCl, 10 mM MgCl_2_, 10 mM DTT, 17 U RiboLock RNase Inhibitor, 0.1% β-mercaptoethanol and 0.025 U inorganic pyrophosphatase (Ppase). *E. coli* σ^70^ RNA polymerase holoenzyme (RNAP) was added and samples were incubated for 7 min following which 5 nM DNA was added for another 7 min. Next, 0.15 mM of GTP, ATP and CTP, 0.015 mM of UTP and 0.8 µCi α^32^P-UTP were added and reactions were run for 15 min. 17 µl of stop solution (95% formamide, 0.5 M EDTA, 0.05% bromophenol blue) were added and samples were denatured for 10 min at 95^0^C prior to loading on a 6% polyacrylamide gel.

### Bioinformatics

Promoter searches were performed using PromScan bioinformatic program (http://molbiol-tools.ca/promscan/). Terminator hairpin was predicted and drawn using MFOLD program (http://mfold.rna.albany.edu/).

## Results

### p_at_ promoter activity is inhibited by the Axe-Txe protein complex

**Figure 1:**
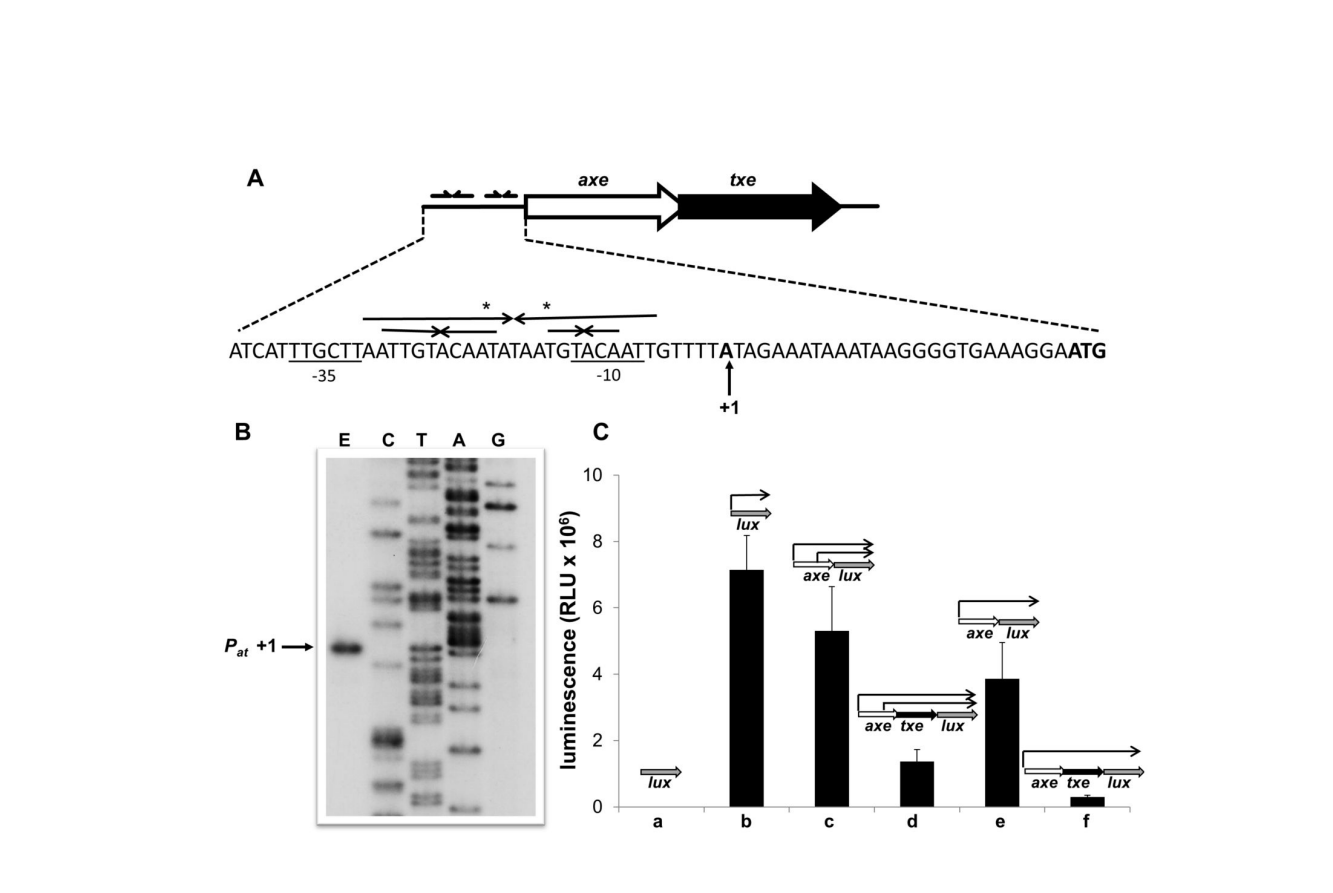
*P*_*at*_ promoter sequence and activity. (**A**) Nucleotide sequence of the *p*_*at*_ region. The transcription start site mapped by primer extension is marked by a vertical arrow. -10 and -35 promoter motifs are underlined and the *axe* start codon is in bold. Palindromes potentially recognised by Axe-Txe are denoted by inverted horizontal arrows. (**B**) Primer extension analysis of *axe-txe* module. Total RNA from *E. coli* SC301467 cells harbouring a plasmid possessing the *axe-txe* operon was subjected to primer extension analysis (E) using a radioactively labelled primer that anneals within flanking vector sequences. Reactions were performed and analysed as outlined in Materials and Methods, and electrophoresed on a denaturing 6% polyacrylamide gel in parallel with nucleotide sequencing reactions (A, C, G, T) carried out with the same primer. The major product from the primer extension is marked as +1. (**C**) Autoregulation of *axe-txe* expression by Axe and Axe-Txe *in cis*. Transcriptional fusions of different fragments of the *axe-txe* operon to the *luxCDABE* operon in pBBRlux-amp plasmid were transformed into *E. coli* SC301467. Luminescence in RLU (relative luminescence units) was measured when cells obtained OD_600_ ~0.4. The results are averages of at least three independent experiments.

Type II TA genes generally are organized in operons and their expression is negatively regulated at the transcriptional level by action of antitoxin alone or in complex with its toxin partner. To assess whether the *axe-txe* genes show a similar scheme of regulation, primer extension analysis was first performed to determine the transcription start point(s) of the *p*_*at*_ promoter. Because it has been shown that the *axe-txe* system is fully functional as a stability cassette in *E. coli* [24], we performed experiments in this bacterium. A single major primer extension product was detected (Figure 1B). Sequences with close matches to consensus -10 (5/6 matches) and -35 (3/6 matches) boxes separated by an optimal 17 bp are located 5’ of this transcription start site (Figure 1A). In addition, a sequence resembling the ribosome binding site (5’-AAGGGG-3’) located 8 nt upstream of the *axe* start codon was observed (Figure 1A).

To assess the influence of Axe and Txe proteins on *p*_*at*_ promoter activity, *in vivo* and *in vitro* tests were performed. A fragment encompassing the *p*_*at*_ promoter and *axe* start codon was inserted upstream of a promoterless *lux* operon in the transcription fusion vector pBBRlux-amp and established in strain SC301467, which is deleted of five chromosomal toxin-antitoxin cassettes [31] to reduce any possible cross interactions from *E. coli* chromosomal TA cassettes, including the *yefM-yoeB* system which is homologous to *axe-txe*. This fusion produced ~7 x 10^6^ RLU, whereas pBBRlux-amp alone produced ~100 units (Figure 1C, bars a and b). Thus, the region 5’ of *axe-txe* possesses a strong promoter activity. In fact, cloning this region upstream of the *lac* operon in different vectors was unsuccessful, generating mutations in the promoter sequence which is a feature characteristic of very strong promoters. To compare the strength of *p*_*at*_, a related promoter of the *yefM-yoeB* system of *E. coli* [10,34] was also cloned upstream of the promoterless *lux* operon in the same vector. This construct produced ~3.5 x 10^5^ RLU. Thus, *p*_*at*_ appears to be a particularly strong promoter.

The 3’ end of *axe* overlaps the 5’ end of *txe* by 8 nt. We aimed to examine the influence of Axe and Txe on *p*_*at*_ activity *in trans* by cloning these overlapping genes under several different arabinose- or IPTG-inducible promoters. Despite many trials, we were not able to clone these genes (data not shown). As an alternative, it was decided to construct *in cis* fusions in which the *p*_*at*_ promoter, followed by *axe* or *axe-txe* genes, was fused to the *lux* operon. In this system, Axe alone inhibited *p*_*at*_ weakly (Figure 1C, bar c) whereas an ~5-fold decrease in *p*_*at*_ activity was observed in the presence of the Axe-Txe complex (Figure 1C, bar d).

**Figure 2:**
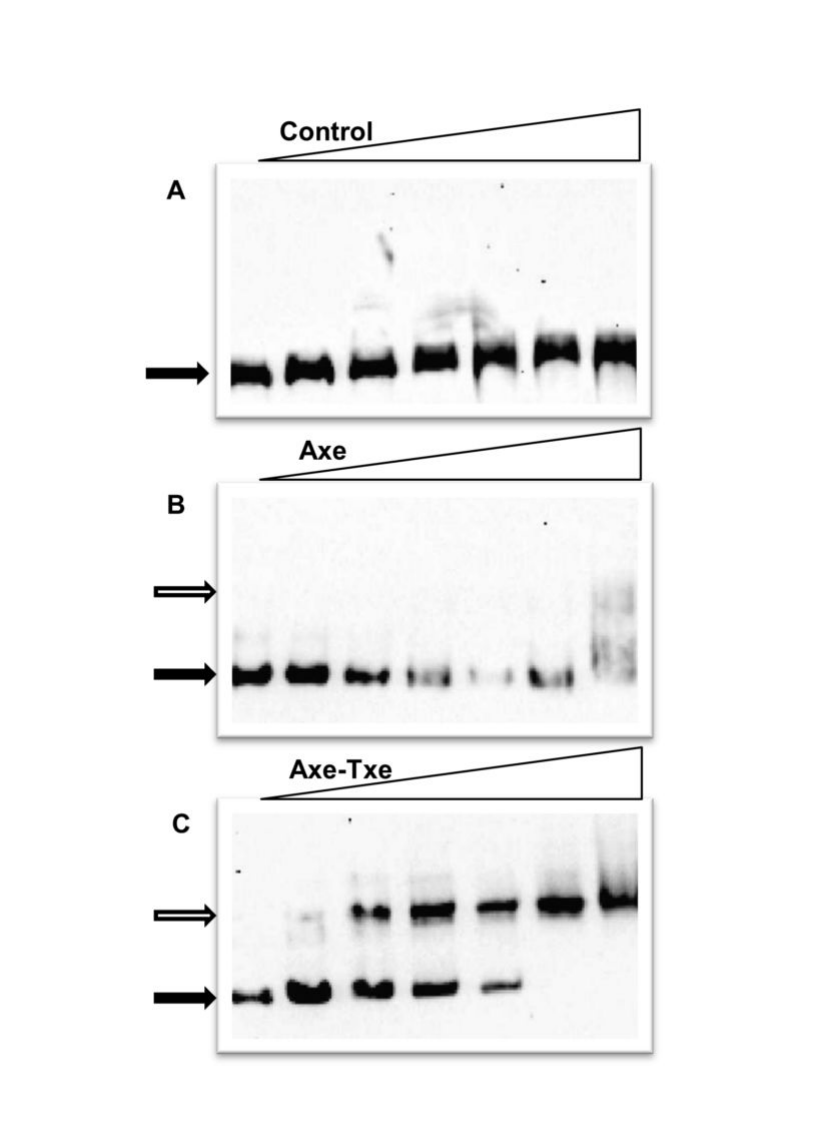
Axe and Axe-Txe binding to the *p*_*at*_ promoter-operator region. A 295-bp 5’ biotinylated fragment that included the *axe* translation start codon and upstream promoter-operator region was subjected to EMSA. The fragment was incubated with different concentrations of *E. coli* BL21(DE3) crude extracts (left to right in each panel): 0, 1.25, 2.5, 5, 10, 12.5 and 25 µg/ml. Reactions were incubated for 20 min at 22^0^C, analyzed by native 5% PAGE, and processed further as outlined in Materials and Methods. (**A**) no Axe or Txe produced; (**B**) Axe overproduction; (**C**) Axe-Txe overproduction. Filled and open arrows denote positions of unbound DNA and protein-DNA complexes, respectively.

Sequence analysis of the *p*_*at*_ promoter region previously revealed two inverted 5’-TGTACA-3’ repeats that are identical to those present in the promoter of the homologous *yefM-yoeB* module and which are responsible for binding the toxin-antitoxin complex [10,34]. Moreover, in the case of *p*_*at*_, these repeats are additionally organized as a more extended inverted repeat with a single mismatch (Figure 1A). These sequences are candidate contact sites for the putative DNA binding N-terminal domain of the Axe antitoxin. To test the affinity of Axe and the Axe-Txe complex for binding to the promoter region *in vitro*, EMSA experiments were performed. For these experiments, BL21(DE3) crude extracts with overproduced Axe or Axe-Txe complex from the pET22(b) vector were used. BL21, like other *E. coli* B strains, does not possess the chromosomal *yefM-yoeB* cassette, thus any potential cross-talk between these two homologous systems can be excluded [35]. Note that cloning of the *axe-txe* genes under the *p*_*T7*_ promoter was possible only if the *p*_*at*_ promoter was included. A 295 bp biotin-labeled fragment containing the promoter region was incubated with different concentrations of crude extracts. Axe alone bound to the promoter fragment only at high extract concentrations (Figure 2B), whereas the Axe-Txe complex retarded migration of the target fragment at lower concentrations of extract, producing one major shifted species (Figure 2C). An extract lacking both proteins did not retard the promoter fragment (Figure 2A). In summary, *in vivo* and *in vitro* experiments indicate that Axe has a weak affinity to the *p*_*at*_ promoter region. In contrast, the Axe-Txe complex binds *p*_*at*_ efficiently *in vitro* and also represses the promoter more effectively than Axe *in vivo*, although this negative regulation of *axe-txe* transcription may be less effective than in other TA systems*.*

### An active promoter which contributes to Txe toxicity is located within the axe gene

**Figure 3:**
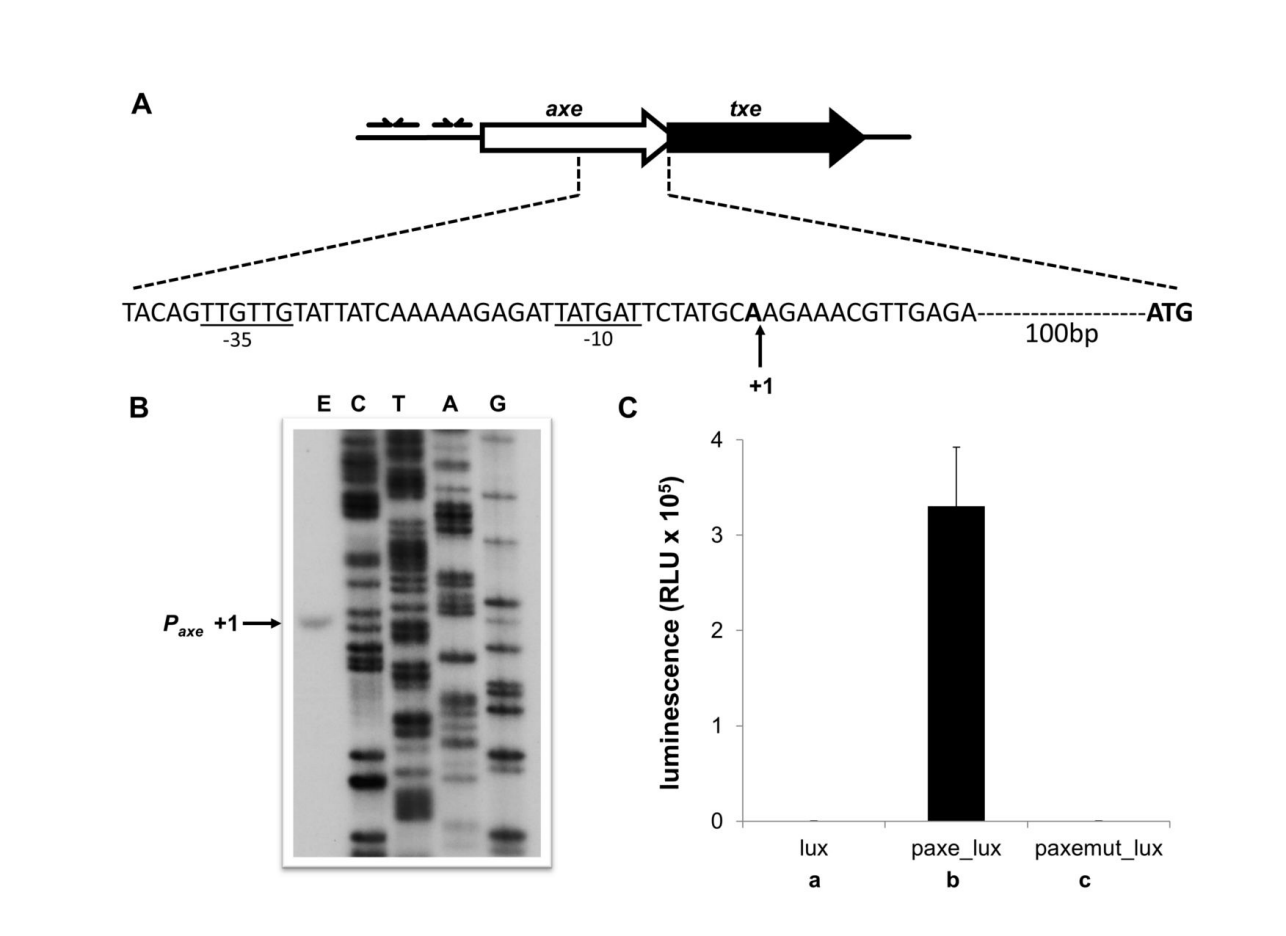
*P*_*axe*_ promoter sequence and activity. (**A**) Nucleotide sequence of the *p*_*axe*_ region. The transcription start site mapped by primer extension is marked by a vertical arrow. -10 and -35 promoter motifs are underlined and the *txe* start codon is in bold. (**B**) Primer extension analysis of *p*_*axe*_. Total RNA from *E. coli* SC301467 cells harbouring a plasmid possessing the *axe* gene was subjected to primer extension analysis (E) using a radioactively labelled primer that anneals within flanking vector sequences. Reactions were performed and analysed as outlined in Materials and Methods, and electrophoresed on a denaturing 6% polyacrylamide gel in parallel with nucleotide sequencing reactions (A, C, G, T) carried out with the same primer. The major product from the primer extension is marked as +1. (**C**) A transcriptional fusion of the *axe* gene to the *luxCDABE* operon in pBBRlux-amp plasmid (paxe_lux) was transformed into *E. coli* SC301467 and luminescence in RLU (relative luminescence units) determined. paxemut_lux denotes a construct in which *p*_axe_ possesses two substitution mutations in the -10 box (see text). The results are the averages of at least three independent experiments.

The inability to clone the *axe-txe* cassette under control of an inducible promoter suggested that regulatory elements additional to *p*_*at*_ might be present in this region. Searches using the PromScan program revealed the presence of a putative promoter within *axe* that might be implicated in expression of the downstream *txe* gene. A fragment of the *axe* gene encompassing this region was fused transcriptionally to the *lux* operon. This fusion produced >3 x 10^5^ RLU confirming the existence of a substantial promoter activity (*p*_*axe*_) within the *axe* coding sequence that might drive expression of *txe* (Figure 3C). This activity was comparable with that obtained for the strong *yefM-yoeB* promoter described above.

Primer extension experiments determined the transcription start point of *p*_*axe*_ (Figure 3B). Sequences with close matches to consensus -10 (5/6 matches) and -35 (3/6 matches) motifs, separated by an optimal 17 bp, are located 5’ of the transcription start site which lies ~110 bp upstream of the translation start codon for the Txe toxin (Figure 3A). To determine if the assigned promoter was responsible for the significant expression observed in the *lux* transcriptional reporter fusion, mutations were introduced into the -10 sequence (TATGAT ->TACGAC) and the mutated sequence (*p*_*axemut*_) was inserted upstream of *lux*. The mutations almost entirely abolished *lux* expression confirming the assignment of *p*_*axe*_ (Figure 3C). EMSA experiments showed that neither the Axe-Txe proteins nor other proteins in the *E. coli* extract bound detectably to a fragment bearing the wild-type p_*axe*_ promoter (Figure S1).

**Figure 4:**
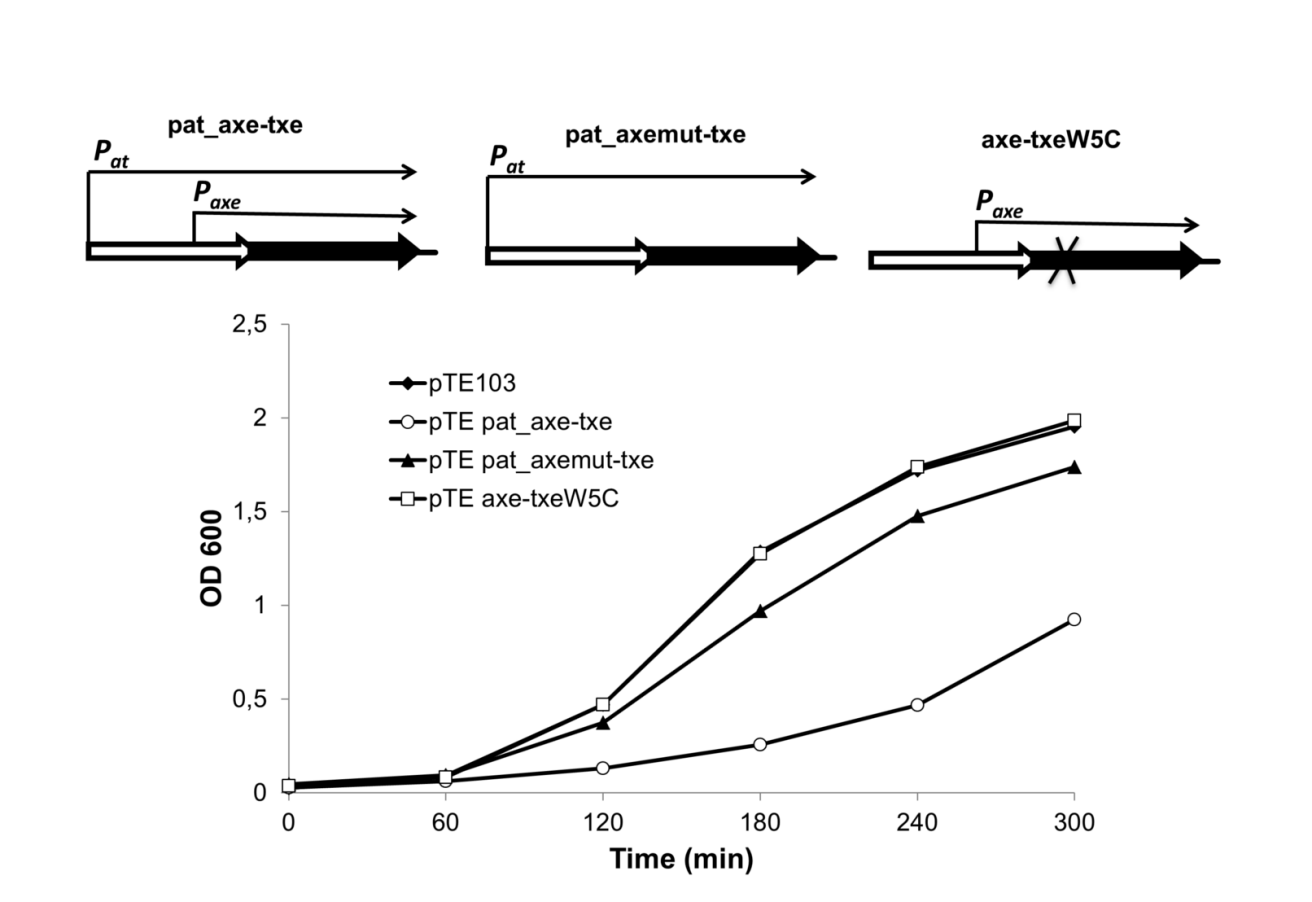
Evidence that *p*_*axe*_ drives the synthesis of Txe toxin. *E. coli* SC301467 harbouring derivatives of pTE103 bearing either the intact *axe*-*txe* module (pTEpat_axe-txe), this cassette in which *p*_*axe*_ was mutated (pTEpat_axemut-txe), or this module producing a nontoxic version of Txe (pTEaxe-txeW5C) were grown at 37^0^C. Absorbance readings at 600 nm were taken at 60 minutes intervals.

The presence of the *p*_*axe*_ promoter internal to the *axe* gene may explain the inability to clone the *axe-txe* cassette under a heterologous promoter: the balance between *axe* and *txe* expression may be altered when *p*_*at*_ is replaced by a different promoter. However, cloning of the *axe-txe* cassette was possible when the *p*_*at*_ promoter was retained at its normal location. Nevertheless, this construct (pTE*pat*_*axe-txe*) inhibited bacterial growth, indicating that *axe-txe* expression was also perturbed (Figure 4). Evidence that *p*_*axe*_ drives the synthesis of Txe was provided by experiments with a strain bearing a plasmid in which the entire *axe-txe* cassette, including the *p*_*at*_ promoter, was again cloned, but in which *p*_*axe*_ carried the -10 box mutations described above (pTE*pat*_*axemut-txe*). These mutations do not change the amino acid sequence of Axe. The growth profile of the strain bearing this plasmid was very similar to strains with either the vector alone or with a plasmid producing a nontoxic version of Txe which also alleviated toxicity (pTE*axe-txeW5C*) (Figure 4). Thus, the *p*_*axe*_ promoter is critical for the toxicity phenotype in this test suggesting that this internal promoter within *axe* is required for *txe* expression.

As described above, *in cis* fusions in which the *p*_*at*_ promoter followed by *axe* or *axe-txe* was fused to the *lux* operon were used to assess repression of this promoter by Axe and Axe-Txe. The data showed that *p*_*at*_ is down-regulated weakly by Axe and more fully by the Axe-Txe complex, although not to basal levels (Figure 1C). To examine any contribution from *p*_*axe*_ in this system, *in cis* fusions were designed in which this promoter was inactivated by the TATGAT ->TACGAC mutations in its -10 box. Reporter data showed that expression levels of *p*_*at*_ in the presence of either Axe alone or Axe-Txe were lower in comparison to those when *p*_*axe*_ is intact (Figure 1C, bars e and f compared to bars c and d). Thus, *p*_*axe*_ contributes significantly to expression levels when wild-type *axe* or *axe-txe* is fused to the *lux* operon, but this expression may not be subject to Axe-Txe regulation. These results also demonstrate that enough *txe* is expressed from *p*_*at*_ alone to produce sufficient levels of Axe-Txe complex for repression of the *in cis* fusion in which *p*_*axe*_ is mutated.

### Active p_axe_ promoter is necessary for proper functioning of the axe-txe cassette as a plasmid stabilization module

**Figure 5:**
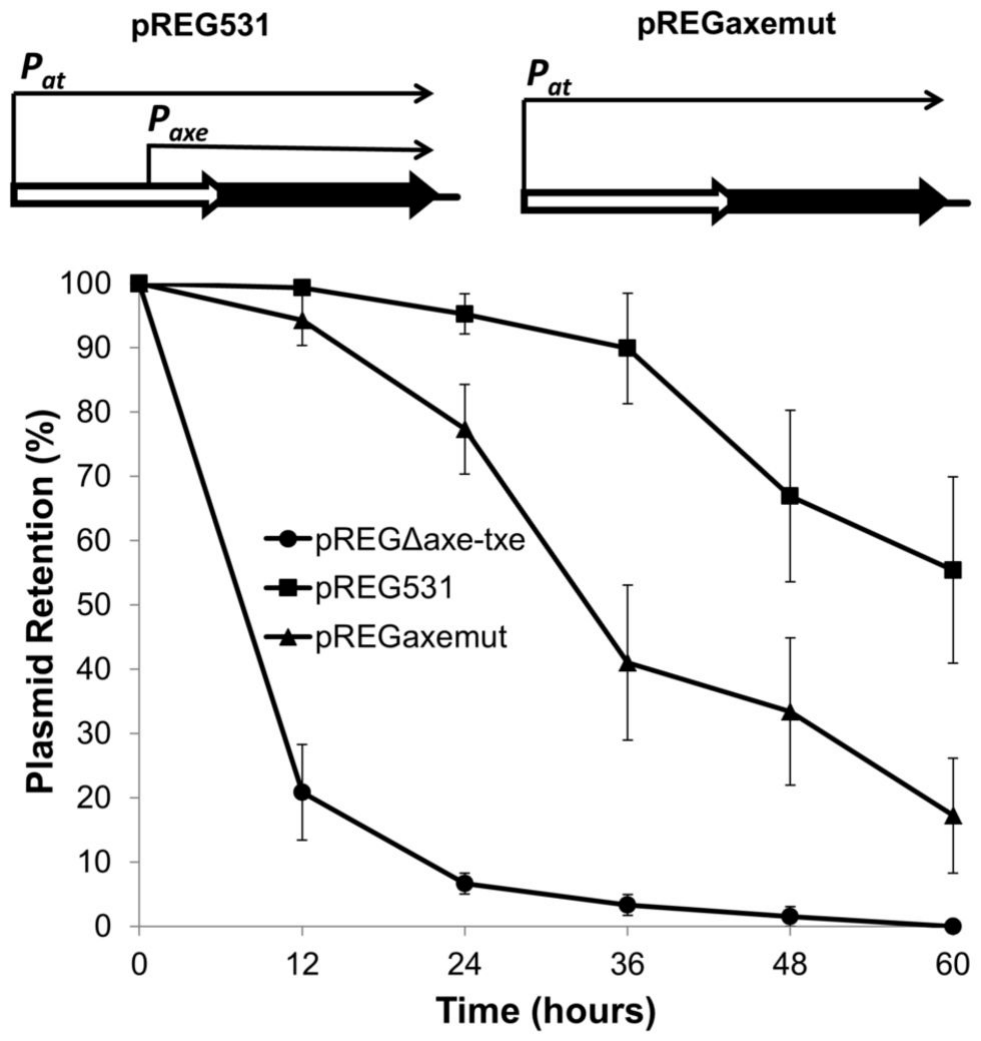
An active *p*_*axe*_ promoter is required for *axe-txe* mediated stable plasmid maintenance. Stability assays were conducted with derivatives of the stability probe vector, pFH450: pREGΔaxe-txe does not contain any accessory stability determinants (circles), pREG531 contains the *axe-txe* cassette (squares), and pREGpaxemut contains the *axe-txe* cassette with a mutated *p*_*axe*_ promoter (triangles). Assays were performed as outlined in Materials and Methods. Results are averages of at least five experiments for which the standard deviation did not exceed 15%.

The major role of toxin-antitoxin cassettes located on plasmid DNA is stable maintenance of these mobile genetic elements in bacterial populations through a post-segregational killing mechanism. Previously, the *axe-txe* cassette was shown to be a functional plasmid stabilization system in evolutionary diverse bacterial hosts, including *E. coli* [24]. To determine whether the active *p*_*axe*_ promoter is necessary for correct functioning of *axe-txe* as a plasmid stabilization module, derivatives of the segregational stability probe vector pFH450 were used [36]. This plasmid contains both moderate-copy-number ColE1 *ori* and low-copy-number P1 plasmid *ori*. However, replication of pFH450 proceeds only from the latter in a *polA* host. As the vector contains no accessory stabilization sequences, it is unstable in this host. Plasmid pREG531 that contains *axe-txe* genes and flanking sequences cloned into pFH450 was used as a positive control [24]. Changes that inactivated the *p*_*axe*_ promoter without altering the Axe amino acid sequence (TATGAT ->TACGAC) were introduced by site-directed mutagenesis producing pREGpaxemut. For the negative control, the *axe-txe* cassette was deleted from pREG531 to produce pREGΔaxetxe. In the absence of antibiotic selective pressure, faster plasmid loss was observed in *E. coli* C600*polA1* bearing pREGpaxemut relative to the strain bearing pREG531 with the wild-type *axe-txe* module (Figure 5). Finally, after 60 hours of discontinuous growth in the absence of selection, plasmid retention for the vector possessing the intact *axe-txe* module was ~55%, whereas the level of plasmid retention was only ~17% for the variant in which the *p*_*axe*_ promoter was inactivated (Figure 5). These results clearly show that the active *p*_*axe*_ is essential for appropriate functioning of the *axe-txe* cassette in stable plasmid maintenance.

### Additional elements within the cassette may influence regulation of axe-txe expression

**Figure 6:**
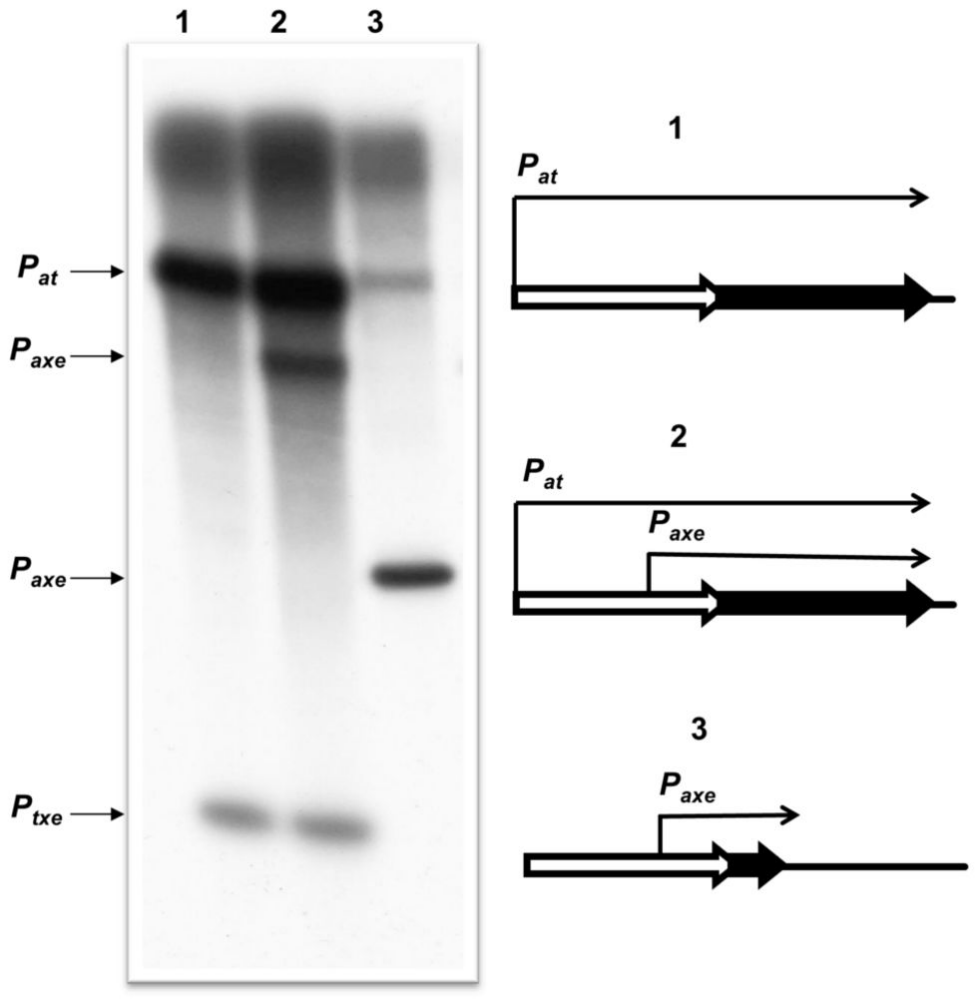
Transcription activity within the *axe-txe* operon. Multi-round *in vitro* transcription experiments were performed using *E. coli* σ^70^ RNA polymerase holoenzyme and pTE103 template DNA containing the whole *axe-txe* operon fragment (2), the same fragment but with the *p*_*axe*_ promoter mutated (1), or the fragment with the *axe* gene and first 60 base pairs of the *txe* gene (3). The band marked as *p*_*txe*_ corresponds to the transcript which derives from as yet unidentified *p*_*txe*_ promoter. Reactions were performed and analysed as outlined in Materials and Methods. Transcript sizes were estimated according to an RNA ladder (RiboRuler Low Range RNA Ladder – Thermo Scientific) which was electrophoresed with the reactions and then excised and stained with ethidium bromide.

*In vitro* transcription analysis of the cassette was performed in the search for regulatory elements that potentially influence expression of the *axe-txe* operon. For this purpose pTE103 plasmid derivatives which contain a strong T7 early transcriptional terminator region were used. Thus, transcripts terminate ~280 bp downstream of the cloned fragments. Transcripts of ~850 and ~680 nt were detected that correspond to those expected to be produced from the *p*_*at*_ and *p*_*axe*_ promoters, respectively (Figure 6, lane 2). Mutation of the -10 box in *p*_*axe*_ abolished production of the smaller transcript which correlates with data presented above that *p*_*axe*_ is a *bona fide* promoter that is required for *txe* expression (Figure 6, lane 1). In addition, these *in vitro* transcription experiments unexpectedly revealed the presence of a third transcript (~300 nt) which appeared only when the whole *txe* gene fragment was present (Figure 6, lanes 1 and 2), but not when a construct with a truncated *txe* gene was employed (Figure 6, lane 3). These observations suggest that this transcript must originate within the *txe* gene.

**Figure 7:**
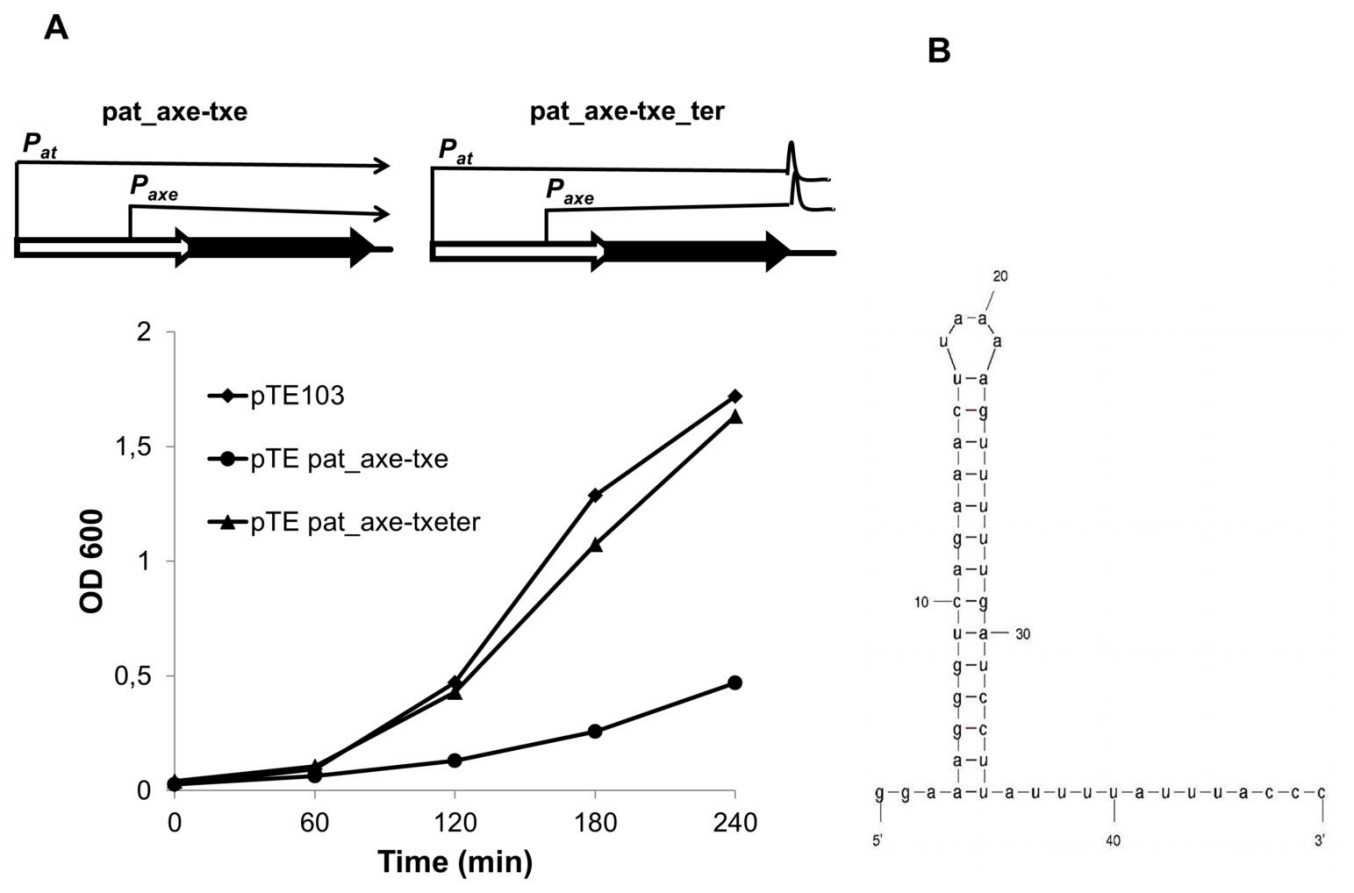
The role of a putative terminator region downstream of the *txe* gene. (**A**) *E. coli* SC301467 harbouring derivatives of pTE103 bearing the *axe-txe* cassette with (pat_axe-txe_ter) or without (pat_axe-txe) the putative downstream transcription terminator were grown at 37^0^C. Absorbance readings at 600 nm were taken at 60 minutes intervals. (**B**) The terminator in the region downstream of the *txe* gene was predicted and drawn by the MFOLD program.

**Figure 8:**
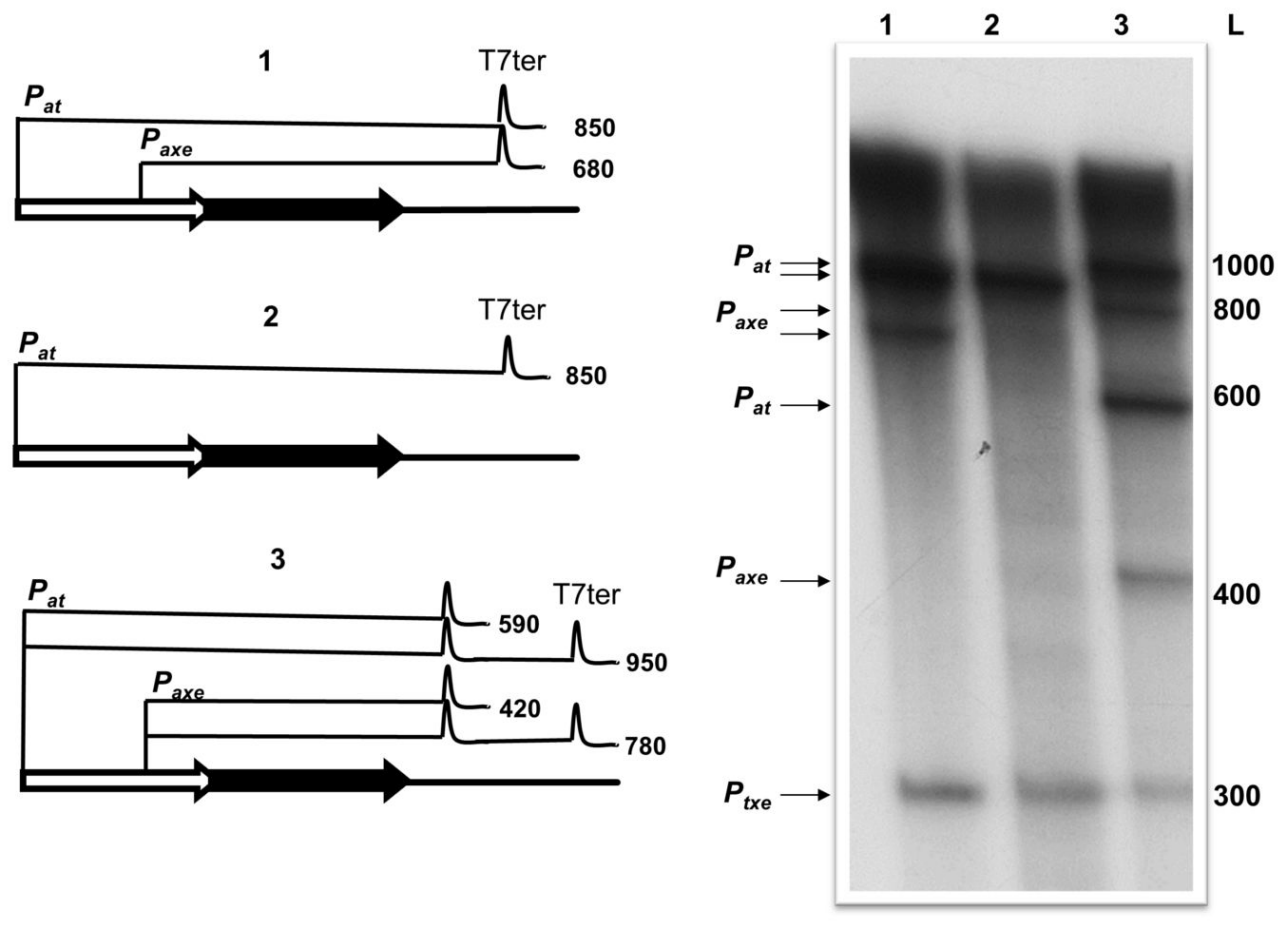
A fragment downstream of *txe* acts as a putative transcriptional terminator/attenuator *in vitro*. Multi-round *in vitro* transcription experiments were performed using *E. coli* σ^70^ RNA polymerase holoenzyme and pTE103 template DNAs containing the whole *axe-txe* operon fragment (1), the same fragment but with the *p*_*axe*_ promoter mutated (2), or the whole *axe-txe* operon fragment plus the downstream putative terminator region (3). Reactions were performed and analysed as outlined in Materials and Methods. Transcript sizes were estimated according to an RNA ladder (RiboRuler Low Range RNA Ladder – Thermo Scientific) which was electrophoresed with the reactions and then excised and stained with ethidium bromide. Positions corresponding to the RNA ladder bands are marked at the right site of the autoradiogram (L). Sizes and schematic representation of the transcripts with the terminator hairpins (“peaks”) are drawn on the left site of the figure.

Comparison of cultures harbouring plasmid pTE103 containing either the complete *axe-txe* module (pTE*pat*_*axe-txe*) or this module with a longer downstream sequence (pTE*pat*_*axe-txe-ter*) revealed significant growth differences (Figure 7A). In the first construct, the region downstream of *txe* comprises ~30-bp after the stop codon. In the second construct ~90-bp longer fragment was included. As observed previously (Figure 4), the construct with short downstream sequences partially inhibited growth due to the expression of *txe* from *p*_*at*_ and *p*_*axe*_ promoters. However, addition of the extended fragment downstream of *txe* alleviated this toxic effect (Figure 7A). Analysis of the sequence revealed the presence of a lengthy transcription terminator-like region starting ~20 bp downstream of the *txe* gene (Figure 7B). *In vitro* transcription assays with constructs bearing the *axe-txe* cassette with this stem-loop fragment showed that it functions as a transcriptional terminator/attenuator *in vitro*. Some of the transcripts deriving from *p*_*at*_ as well as from *p*_*axe*_ promoters stop at this point, while the rest terminate further at the T7 strong terminator located within the vector (Figure 8, lane 3). This putative hairpin structure may have a role in transcript stability if it is recognized by RNases that decrease the stability of the mRNAs and thereby modulate Txe production. This hypothesis is being tested currently. Moreover, the *axe-txe* cassette without this potential terminator region cloned into a stability probe vector clearly showed impaired activity as a stability determinant indicating the importance of this element, possibly to ensure an optimal stoichiometry between toxin and antitoxin (unpublished data).

## Discussion

The toxin components of TA systems are intracellular molecular time bombs whose release from complexes with their cognate antitoxins can trigger bacterial programmed cell death or cell cycle arrest [5]. Understanding the mechanisms by which expression and activation of these modules are controlled is crucial to dissect their functioning and possible practical exploitation.

The Axe-Txe system was first discovered on the multidrug-resistant pRUM plasmid in a clinical isolate of *E. faecium* [24]. Preliminary analysis of Axe–Txe demonstrated that it functions as a characteristic TA system: expression of Txe is toxic to cells, Axe alleviates Txe-induced toxicity, and Axe–Txe increases plasmid maintenance [24]. It was also demonstrated that Txe is an endoribonuclease which cleaves mRNA and thereby inhibits protein synthesis [27]. Due to the prevalence of the *axe–txe* genes on plasmids in enterococcal isolates [29,30], artificial activation of Txe presents an attractive antimicrobial strategy. However, a complete lack of knowledge about regulation of *axe-txe* expression blocks potential exploration of the complex as an antimicrobial target.

The chromosomal *yefM-yoeB* toxin-antitoxin module of *E. coli* is homologous to *axe-txe* [24]. As is the case with most known TA systems, expression of *yefM-yoeB* is negatively autoregulated, with YefM being the primary transcriptional repressor and YoeB acting as a repression enhancer [10]. DNA binding is achieved by the sequential association of YefM with a pair of inverted repeats that comprise the *yefM-yoeB* operator site [10]. This interaction involves a pair of arginine residues in a unique DNA binding fold within the N-terminal region of the protein [34,35]. The YoeB toxin acts as a corepressor by stabilizing the flexible C-terminal region of YefM which also conceals the toxin’s endoribonuclease fold [35].

Analysis of the nucleotide sequence of the *p*_*at*_ promoter-operator region upstream of *axe-txe* revealed two inverted repeats with the same 5’-TGTACA-3’ core that overlap the *yefM-yoeB* promoter [10]. In the case of *p*_*at*_, the repression by antitoxin alone was very weak (<2-fold), whereas the Axe-Txe complex repressed more efficiently (~5-fold). However, the activity of the *p*_*at*_ *-lux* fusion remained very high in the repressed state. These results suggested that there might be another mechanism(s) which shut downs *axe-txe* expression. In agreement, an additional promoter (*p*_*axe*_) within the *axe* gene directs extra synthesis of Txe protein. However, this promoter lacks overlapping 5’-TGTACA-3’ boxes, is not repressed by Axe-Txe, and no detectable binding to this region was observed by Axe-Txe *in vitro*. The *p*_*axe*_ promoter instead may be regulated by an unknown factor(s), or may be expressed constitutively. The ~300-nt transcript produced by the *axe-txe* cassette may also be implicated in controlling expression of the *p*_*axe*_ promoter by an unknown mechanism. Nevertheless, the data clearly show that the active *p*_*axe*_ promoter is indispensable for proper functioning of the *axe-txe* cassette as a plasmid stabilization module.

The control of the synthesis of most, if not all, toxin proteins of TA complexes is likely to be multilayered. Further indications that *axe-txe* may be subject to additional levels of regulation came from experiments with fragments containing the *axe-txe* cassette but with different lengths of downstream sequence. Constructs possessing an extended fragment downstream of *txe* that contains a putative terminator region do not inhibit bacterial growth, whereas constructs which lack this fragment exert a pronounced growth defect. One can speculate that the potential termination hairpin may serve as an element that decreases mRNA stability and in this way lowers production of the Txe toxin. mRNA stability is one of the parameters that determine the efficiency of gene expression. mRNA turnover is mediated by a combination of endo- and exoribonucleases whose activities are modulated by structural features of the mRNA [37]. One such example is the *kis-kid* toxin-antitoxin system in which the intracellular levels of Kis and Kid proteins are controlled by limited degradation of a polycistronic messenger. However, in this case the presence of a stem-loop sequence located within the 5’ region of *kid* gene shows a stabilizing effect mediated on mRNA [38]. The majority of RNA molecules are subjected to regulation and, as is the case of mRNA, their decay can be influenced by growth conditions. Moreover, the RNA degradosome can undergo changes in composition depending on growth or stress conditions [39–41].

In the case of *axe-txe* different regulatory mechanisms might exist to ensure a balanced production of the antitoxin relative to the toxin which is necessary for appropriate functioning of this system. The *kis-kid* and *ccdAB* operons are tightly regulated by the ratio of the toxin and the antitoxin [13,14]. It is possible that in the reporter system used here, in which the *axe-txe* operon lacking the terminator-like sequence downstream of *txe* was fused with the *lux* gene, the ratio of Axe and Txe was not optimal for full repression of *p*_*at*_ promoter due to the excess of the toxin arising from altered mRNA stability. This agrees with other data showing that an excess of toxin can abolish transcriptional repression by releasing the TA complex from the operator site [15,16].

It should be emphasized that observations about *axe-txe* regulation presented in this paper are true for *E. coli* and may differ in the natural host, *E. faecium*. On the other hand, study of TA systems that derive from different bacterial species, including *Streptococcus, Staphylococcus,* *Synechocystis* *,* *Streptomyces* and *Vibrio*, in an *E. coli* model is common [42–46]. Nevertheless, studies of *axe-txe* regulation in the natural host will reveal whether different regulatory mechanisms operate in *E. faecium* compared to *E. coli*.

In conclusion, the data presented here show that the regulation of expression of the *axe*-*txe* module appears to be very complex. The *p*_*at*_ promoter activity is very high and is only partially repressed by the concerted action of the Axe-Txe complex. Moreover, another promoter, *p*_*axe*_, provides additional expression of the *txe* gene. Therefore, the expression of the toxin gene requires additional negative regulation. This may be achieved by two means: (i) decreased stability of *txe* mRNA due to its degradation starting after formation of a specific hairpin structure at the 3’ end of the transcript; and (ii) the action of a counter transcript derived from the promoter located within *txe* gene. Our experiments clearly indicate that both the active *p*_*axe*_ promoter and the region downstream of *txe* gene with the putative terminator region are necessary for proper functioning and tight regulation of the *axe-txe* cassette.

One might ask why did such a complicated regulatory system evolve in the *axe-txe* module? We speculate that additional regulatory elements provide more possibilities to optimize toxin and antitoxin production under diverse environmental conditions, e.g., nutrient availability or different temperatures. This may be especially important for bacteria living under conditions with potentially rapid fluctuations, including enterococci occupying the mammalian intestine that are suddenly excreted outside their host in stools. The balance between the amounts of toxin and antitoxin is of particular importance for cell survival.

## Supporting Information

Figure S1 **Neither Axe-Txe proteins nor other proteins in the *E. coli* extract bound detectably to a fragment bearing the wild-type p_*axe*_ promoter.** A 126 bp 5’ biotinylated fragment that includes *p*_*axe*_ was subjected to EMSA. DNA samples were incubated with the different crude extracts concentrations of *E. coli* BL21(DE3) harbouring pET22at_axe-txe plasmid (left to right): 0, 1.25, 2.5, 5, 10, 12.5 and 25 µg/ml for 20 min at 22^0^C and analyzed by a native 5% PAGE. Reactions were processed as outlined in Materials and Methods.
(TIF)
