# Supplementary figures and images for: The Axe-Txe Complex of Enterococcus faecium Presents a Multilayered Mode of Toxin-Antitoxin Gene Expression Regulation
Source: PLoS One. 2013 Sep 3;8(9):e73569. doi: 10.1371/journal.pone.0073569 (PMC3760812; doi:10.1371/journal.pone.0073569)

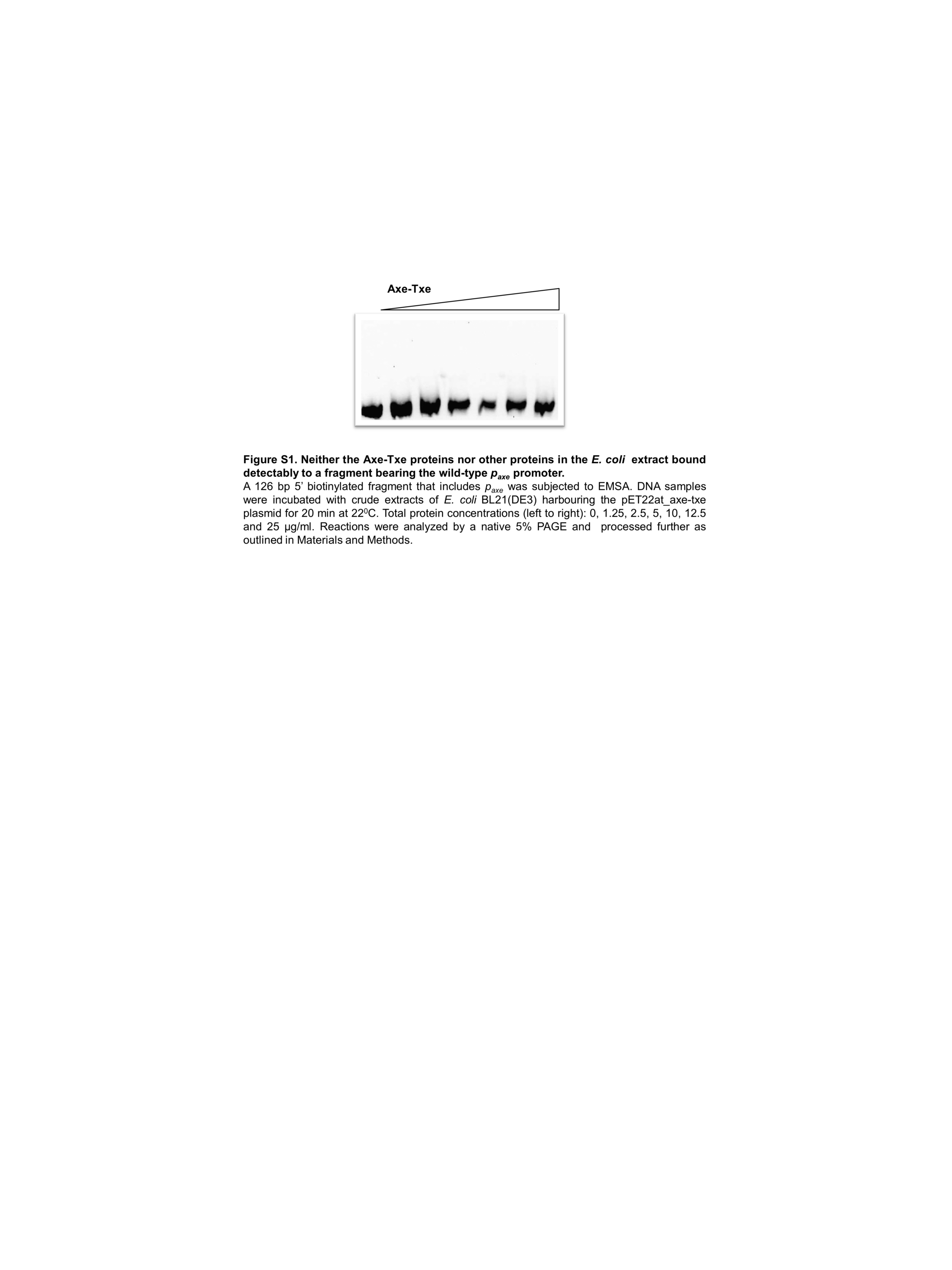

Supplement: Figure S1 — Neither Axe-Txe proteins nor other proteins in the E. coli extract bound detectably to a fragment bearing the wild-type paxe promoter. A 126 bp 5’ biotinylated fragment that includes p axe was subjected to EMSA. DNA samples were incubated with the different crude extracts concentrations of E. coli BL21(DE3) harbouring pET22at_axe-txe plasmid (left to right): 0, 1.25, 2.5, 5, 10, 12.5 and 25 µg/ml for 20 min at 220C and analyzed by a native 5% PAGE. Reactions were processed as outlined in Materials and Methods. (TIF) [file pone.0073569.s001.tif]
